# The UK clinical research network - has it been a success for dermatology clinical trials?

**DOI:** 10.1186/1745-6215-12-153

**Published:** 2011-06-16

**Authors:** Kim S Thomas, Karin Koller, Katharine Foster, Jo Perdue, Lisa Charlesworth, Joanne R Chalmers

**Affiliations:** 1 University of Nottingham, Centre of Evidence Based Dermatology, University of Nottingham, King's Meadow Campus, Lenton Lane, Nottingham NG7 2NR, UK

## Abstract

**Background:**

Following the successful introduction of five topic-specific research networks in the UK, the Comprehensive Local Research Network (CLRN) was established in 2008 in order to provide a blanket level of support across the whole country regardless of the clinical discipline. The role of the CLRN was to facilitate recruitment into clinical trials, and to encourage greater engagement in research throughout the National Health Service (NHS).

**Methods:**

This report evaluates the impact of clinical research networks in supporting clinical trials in the UK, with particular reference to our experiences from two non-commercial dermatology trials. It covers our experience of engaging with the CLRN (and other research networks) using two non-commercial dermatology trials as case studies. We present the circumstances that led to our approach to the research networks for support, and the impact that this support had on the delivery of these trials.

**Results:**

In both cases, recruitment was boosted considerably following the provision of additional support, although other factors such as the availability of experienced personnel, and the role of advertising and media coverage in promoting the trials were also important in translating this additional resource into increased recruitment.

**Conclusions:**

Recruitment into clinical trials is a complex task that can be influenced by many factors. A world-class clinical research infrastructure is now in place in England (with similar support available in Scotland and Wales), and it is the responsibility of the research community to ensure that this unique resource is used effectively and responsibly.

## Background

The last five years have seen an unprecedented increase in support for the conduct of applied clinical research in the UK. The changes introduced as a result of the far-reaching report "Best Research for Best Health"[1], called for the development of the National Health Service (NHS) as a world leader in delivering high-quality and timely clinical research. Through the development of the National Institute for Health Research (http://www.nihr.ac.uk), many changes have now been implemented. Some of the key developments include: the streamlining of research approval processes; the development of a national portfolio of trials to improve accountability and clarity of trial reporting; and the establishment of clinical research networks - with the aim of supporting the delivery of high quality research within the NHS. Topic specific networks have been created in areas of high priority (e.g. stroke, cancer and mental health), and over-arching networks have been created to support research across a variety of disciplines (the Medicines for Children Research Network - MCRN; the Primary Care Research Network - PCRN; and the Comprehensive Local Research Network - CLRN). The overall objectives of these networks are: i) to introduce effective systems to speed up the regulatory approval process; ii) to provide infrastructure to support clinical research activities; and iii) to facilitate access to appropriate patient populations.

This brief paper focuses on the impact that the clinical research networks have had on delivery of trials conducted in the field of dermatology, using two non-commercial randomised controlled trials (RCTs) as case studies.

Dermatology was well-placed to take advantage of the newly emerging research infrastructure as it developed, since the discipline already had a well-developed collaborative research network in the form of the UK Dermatology Clinical Trials Network (http://www.ukdctn.org). This led to dermatology being identified as a priority area in 18 of the 25 CLRN regions in England, many of which were able to provide considerable support in the form of research nurses, trial administrators, and support for the time of recruiting clinicians.

This paper describes the impact that CLRN, MCRN and PCRN support had on the completion of two dermatology trials, and highlights some of the challenges and lessons that we have learnt as a result. These case studies are presented in chronological order based on the time at which support from the Clinical Research Networks was first accessed.

## Methods

### Case Study 1: Softened Water Eczema Trial (SWET)

#### The scenario

The Softened Water Eczema Trial (SWET) was a multi-centre randomised controlled trial funded by the NIHR Health Technology Assessment programme (HTA) [2,3]. The study was well-resourced and included funding for four part-time research nurses and a trial manager. Recruitment was anticipated to take 18 - 24 months starting in March 2007. Despite overwhelming support from patients, recruitment had been slower than anticipated due to changes in personnel (one research nurse left and one went on maternity leave), and a higher than expected numbers of participants' homes that were found to be unsuitable for the installation of a water softener (the intervention).

The Trial Steering Group decided to focus on four options to boost recruitment: i) focussing on media advertising, ii) opening new centres, iii) referring patients from nearby hospitals, and iv) approaching the NIHR Clinical Research Networks for support. In October 2008, the Trial Steering Group approved a request to the funders for a six-month extension to the study, at an estimated cost of £100,000.

#### Involvement of NIHR Clinical Research Network

The SWET trial was adopted by the MCRN and added to the NIHR portfolio of trials in March 2008 [4]. The MCRN provided maternity leave cover from August 2008 for our London centre (which up until this point had been our top recruiting site), and allowed us to open two new recruiting centres in Lincoln (June 2008), and South East London (December 2008).

In addition, Trent CLRN and Hampshire and Isle of Wight CLRN provided nurse time in Nottingham, and administrative support in the Isle of Wight. This additional support meant that the SWET research nurses were able to open up two new recruiting centres in Leicester and Portsmouth respectively.

We also approached the PCRN, who assisted with the identification of suitable patients from GP databases in Cambridge, Isle of Wight and Leicester.

Although the speedy intervention of the research networks allowed us to maintain our recruitment rate at a time when it might otherwise have declined, by far the most influential event in ensuring that the SWET study completed recruitment to target was the appearance of the principal investigator for Portsmouth on the local BBC TV News. This resulted in a deluge of calls to the co-ordinating centre (more than 700 contacts over a period of 2 weeks). Only through the speedy response of the Trent CLRN in providing administrative support for the Trial Manager were we able to respond to these queries, and translate this interest into improved recruitment.

#### Overall impact

Through a combination of the measures outlined above, the recruitment target was reached by June 2009. This meant that the trial was delivered on budget (after a short 4 month no-cost extension for analysis and write-up), and the proposed funding extension of £100,000 was not required.

**Figure 1:**
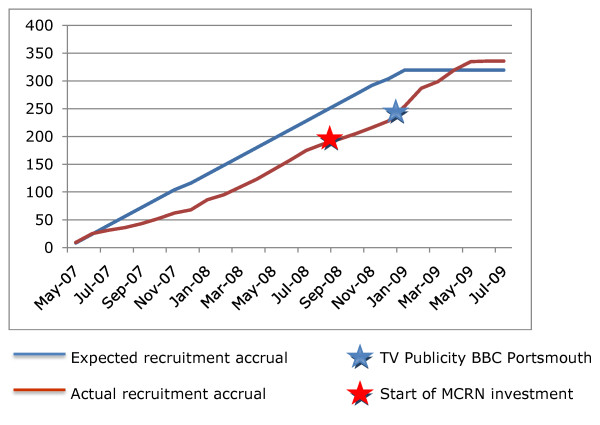
**Recruitment graph for SWET trial**.

The dramatic increase in trial recruitment during the final six months of the trial is illustrated in Figure 1.

### Case Study 2: Prophylactic Antibiotics For The Prevention Of Cellulitis Of The Leg (Patch I Trial)

#### The scenario

The PATCH study was the first trial to be conducted through the UK Dermatology Clinical Trials Network, and was funded by a medical charity (Action Medical Research). This trial was intended to test the ability of the Network to recruit into trials by identifying just a few patients from many centres; thus allowing busy clinicians to engage in research in a meaningful way, without over burdening any one site. As a result funding for the trial was limited to a trial manager and an administrator at the co-ordinating centre, but no dedicated research nurses at recruiting sites. Whether such a model is possible in the current regulatory climate, and for a condition such as cellulitis, which is often seen by specialties other than dermatology, is unclear. Subsequent UK DCTN trials of rare skin conditions that are being treated by dermatologists are proving more successful in this regard (http://www.ukdctn.org).

Recruitment was anticipated to take 12 months from April 2006 to April 2007. However, by Dec 2007, recruitment was just 62 (24%) of target and we were faced with having to close the trial early due to lack of funding. Indeed, a related trial (PATCH II) was closed at this time [5].

At the time of approaching the NIHR CRN, recruitment into the PATCH trial was hampered by three key factors: i) the absence of trial staff at each of the 29 recruiting centres meant that all trial related administrative tasks were devolved to the recruiting clinician, making it very time consuming for busy clinicians; ii) because cellulitis is an acute inflammation of the skin and underlying tissue, patients were generally treated in emergency care settings, rather than on a dermatology wards or outpatients setting. This made it difficult, and time-consuming for our network of volunteer dermatologists to identify and recruit suitable patients; iii) because recruitment during the early phases of the trial had been slow, the supply of trial drugs was due to expire, and funds were not available from the funder to buy further supplies, potentially halting recruitment if alternative arrangements were not found.

#### Involvement of NIHR CRN

The PATCH study was adopted onto the UK CRN portfolio of trials in April 2008. All CLRN areas where dermatology was identified as a priority area were approached with a view to providing local support at recruiting sites (either research nurses or trial administrators) to reduce the burden on the dermatologists. In total, eight CLRNs provided additional support, ranging from 1 day a week of research nurse time, to full time nurses or administrators.

In addition, the PCRN provided assistance with identifying patients through GP surgeries in one region, and the Trent CLRN agreed to cover the costs of purchasing more trial drugs using money that had been ring-fenced for "unblocking the blocks in pharmacy".

However, for the PATCH trial (as with the SWET trial), the biggest boost to recruitment came when we advertised for patients using local media (radio and adverts placed in local papers). This approach resulted in a substantial boost to recruitment (>50% increase in recruitment rate), but again resulted in a considerable administrative burden at the co-ordinating centre. All calls were screened by staff at the co-ordinating centre, before being referred to the relevant recruiting hospital. In order to meet this challenge, Trent CLRN provided funding for a full-time trial administrator.

#### Overall impact

**Figure 2:**
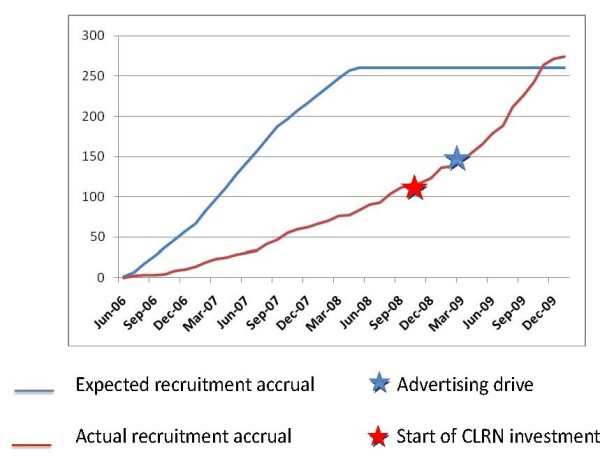
**Recruitment graph for PATCH I Trial**.

Once again, it was the combined approach of various strategies, coupled with an understanding of the importance of advertising the trial beyond the traditional clinical setting that resulted in a positive outcome for the PATCH trial. By having increased support at the recruiting centres, the benefits of a centrally co-ordinated publicity drive were immediately translated into improved recruitment rates at centres throughout the UK. The study was closed to recruitment in January 2010, having exceeded its target of 260 participants (Figure 2).

Both of the trials described in this paper were ongoing at the time that resources became available through the NIHR Clinical Research Networks, which meant that the role of the research networks in these trials was primarily "troubleshooting". Future trials will work more collaboratively with the research networks, and will benefit greatly from both the systems and the personnel that are now in place. This was demonstrated clearly with another of our dermatology trials that was adopted onto the UK CRN portfolio more recently (the BEEP feasibility study - Barrier Enhancement for Eczema Prevention) [6]. In this case, support was available from the outset from both the CLRN and MCRN, and the trial quickly recruited in excess of its target in three hospitals across the region.

## Discussion

The introduction of research networks with dedicated nursing and administrative support for clinical trials has been a fantastic boost to the delivery of clinical research within the NHS. Nevertheless, this paper provides a useful forum in which to highlight some of the key concerns that researchers face.

### How should success be measured?

As with all new initiatives, it is important to have useful metrics with which to measure success. To date, one of the main ways of doing this has been an evaluation of recruitment rates. Whilst there has been much talk about the need to recognise the variability of study design in summarising recruitment rates, it is still not entirely clear how this is achieved. In the absence of transparency, it is difficult to interpret summary graphs that combine epidemiological studies (involving potentially thousands of participants) with RCTs in rare conditions (where recruitment of 1-2 participants per centre may be happening on a global scale). In addition, future metrics should include additional important measures such as recruitment to time/target, completion of follow-up, low attrition rates, and timely publication of trial findings. These areas are just as important as overall trial recruitment if findings are to improve the lives of patients and inform clinical decision making. In this regard, we have been particularly helped by the Trent CLRN, which currently provides support for two dermatology trial administrators. These posts not only support recruitment into our trials, but also play a big role in ensuring ongoing data collection, participant retention and trial completion.

Of particular relevance to the Comprehensive Local Research Networks is the need to ensure that there is appropriate transparency and accountability across the different networks. As for many disciplines, dermatology spans many topic areas and many of our trials involve collaboration with the Medicines for Children Research Network (MCRN), the Primary care Research Network (PCRN), and the Cancer Research Network (CRN), as well as the generic CLRN. Ensuring that all networks are adequately recognised in contributing to the success of a study can be a challenge, and relies upon correct tagging of the trials as they are entered onto the portfolio. In our experience, this is an area that can be facilitated by the close involvement of the relevant Specialty Groups [7], so that recruitment figures are credited appropriately across multiple networks. For example, a recent audit of the portfolio by the Dermatology Specialty Group found four skin cancer trials that were included in the cancer network's portfolio but were not in the dermatology portfolio (even when the lead investigator on the trial was a dermatologist). By writing to all of the relevant investigators, and in close collaboration with the portfolio teams at the Clinical Research Network Co-ordinating Centre, this situation was rectified and recruitment figures were collected by all relevant networks.

### Does the system discourage collaborative, multi-centre trials?

Given that future funding is tied to current activity by CLRN region, there is a possibility that investigators will be tempted to design trials that recruit solely within their own region in order to secure future infrastructure support. This has potential to reduce the generalisability of trial results if participants all come from a very select population or region.

For an organisation such as the UK Dermatology Clinical Trials Network that specialises in conducting trials in rare conditions, individual centres may contribute just one or two participants per year. It is possible that local Trusts and investigators may find studies of this kind less appealing than ones that are able to make a larger contribution to their overall recruitment rates. Future adoption of trial findings may be impeded if fewer clinicians have had experience of using and prescribing the newly investigated products [8].

### Administration and implementation

As with any new system, it takes time for the new administrative systems and financial arrangement to be streamlined. However, our experiences to date would suggest that there is still room for improvement in this regard.

For multi-centre trials that require support at a national level, negotiating local arrangements can be time-consuming and potentially fragmented. Further streamlining of the application process in line with other regulatory approvals in order to avoid duplication of effort is required. Indeed, this is now emerging and all new studies are assessed for adoption onto the NIHR portfolio at an early stage, and a designated "lead network" is identified as part of the application process through the NIHR Coordinated System for gaining NHS Permissions (http://www.crncc.nihr.ac.uk/about_us/processes/csp). This process ensures that the needs of studies are evaluated prior to initiation of the research activity, and negotiations with other networks are facilitated by experienced network staff familiar with the process.

In addition, fostering and enhancing shared working practices between NHS Trusts and the Universities, where much of the research is planned and managed, is still a crucial area. It is important that systems created in order to generate and facilitate high quality research within the NHS allow for close collaboration between NHS and University colleagues.

Managing the day-to-day activities of research staff employed through the CLRN can also be challenging. The needs of these staff are unique in that they are constantly working with different research teams and are involved in the delivery of multiple trials across the portfolio. This role can be less satisfying for the individual research nurses/administrators as their role is often seen as being one of "plugging the gaps" rather than as an integrated and valued member of the research team. This can lead to high staff turn-over and engagement with the individual studies could be compromised.

Equally, for managers the role can be difficult and time-consuming. Responsibility for deciding which studies are to receive support, allocating tasks, training and managing the staff, negotiating hours, and planning for future activity requires a broad understanding of both research design and the needs of local teams.

## Conclusion

The last five years have seen unprecedented support for clinical research in the UK and one which has transformed the way in which research is designed and implemented in this country. The additional resource allocated to the trials highlighted in this report meant that both studies recruited to target and that two important clinical questions can now be addressed. However, it is clear that simply increasing resources is not sufficient in isolation. Neither of the trials would have benefited from the increased financial support without the availability of experienced and extremely dedicated research teams, who strove to ensure that the trials were of high quality and delivered to target. Success was driven by a genuine partnership between the research teams and the flexibility of network support.

For both trials, raised public awareness was key. Further input into the ways in which members of the public can be made aware of ongoing trials, as well as initiatives to improve the public's understanding of clinical research would be beneficial.

The next challenge for researchers is to find novel and innovative ways of ensuring that new research is implemented into practice, and to look at ways of building treatment evaluation into normal clinical practice. This will be facilitated if the newly evolving structures allow easy and transparent support across the whole country, rather than encouraging competition between geographical regions.

## Competing interests

All authors have completed the Unified Competing Interest form at http://www.icmje.org/coi_disclosure.pdf (available on request from the corresponding author) and declare that they have no involvement with any companies relevant to this submission.

## Authors' contributions

KT contributed to the design, conduct, and analysis of the two trials contained in this paper, wrote the first draft of this article, and approved the article for submission. KF, JP, LC contributed to the conduct of the two trials contained in this paper, commented on drafts of this article, and approved the article for submission. KK contributed to the design, conduct and write-up of the SWET trial contained in this paper, commented on drafts of this article, and approved the article for submission. JC contributed to the design, conduct and write-up of the PATCH trial contained in this paper, commented on drafts of this article, and approved the article for submission.

## Authors' information

KT Associate Professor; KK Trial Manager; KF Trial Manager; JP CLRN Trial Administrator; LC CLRN Trial Administrator; JR Research Fellow at Centre of Evidence Based Dermatology, University of Nottingham.
